# Dynamic blood flow imaging with ^99m^Tc-hydroxymethylene diphosphonate as a therapeutic response marker in patients with Raynaud’s phenomenon

**DOI:** 10.1038/s41598-023-47197-3

**Published:** 2023-11-13

**Authors:** Jang Yoo, Miju Cheon

**Affiliations:** Department of Nuclear Medicine, Veterans Health Service Medical Center, Seoul, South Korea

**Keywords:** Biomarkers, Rheumatology, Rheumatic diseases

## Abstract

We evaluated the predictive value of dynamic blood flow scintigraphy with ^99m^Tc-HDP (hydroxymethylene diphosphonate) for therapeutic response in patients with Raynaud’s phenomenon (RP). Eighty patients who underwent dynamic blood flow scintigraphy using the one-hand chilling method were enrolled. We analyzed the quantitative variables as the ratio of chilled fingers to ambient fingers (CA_finger_), that of the chilled hand to ambient hand (CA_hand_), and that of chilled fingers to ambient palm (FPR) (CA_FPR_) at 15 and 30 s after ^99m^Tc-HDP bolus injection. Total cumulative radioactivity counts for 180 s were obtained. We evaluated the clinical utility of these quantitative parameters with other clinical variables, including RP severity, therapeutic compliance, types of RP, and scintigraphic interpretation of findings in patients with RP. Fifty-two patients showed poor therapeutic response. There were significant differences between good- and poor-therapeutic responder groups in RP intensity (p = 0.003), CA_finger15s_ (p = 0.008), CA_finger30s_ (p = 0.002), CA_finger180s_ (p = 0.011), CA_hand15s_ (p = 0.008), CA_hand30s_ (p = 0.007), CA_hand180s_ (p = 0.017), CA_FPR30s_ (p = 0.004), and CA_FPR180s_ (p = 0.002). After multivariate logistic regression analysis, only CA_finger30s_ (p = 0.002) had an independent predictive value of the therapeutic response. ^99m^Tc-HDP dynamic blood flow scintigraphy could be helpful in predicting the therapeutic response in patients with RP.

## Introduction

Raynaud’s phenomenon (RP) is a clinical manifestation defined by paroxysmal reversible episodes of vasospasm in the peripheral vascular system of distal fingers or toes, resulting in a cold sensation, color changes, pain, and paraesthesia^1, 2^. RP is classified into two types based on the underlying etiology. Primary RP, also named idiopathic RP, means no evidence of an underlying disorder exists. Although the pathogenesis of primary RP has not been fully clarified, several studies demonstrated that hypersensitivity and dysregulation of the autonomic nervous system can be considered the predominant factor in primary RP pathogenesis^3, 4^. Primary RP has several features, such as young age of onset (usually before 30 years of age), the presence of a family history of RP, symmetrical manifestation, negative results of antinuclear antibody (ANA) or their presence of low titers, and the absence of any other auto-antibody laboratory findings. Secondary RP is diagnosed when it is associated with underlying disorders such as peripheral arterial disorder (e.g., obstructive vascular disease), systemic connective tissue disease (e.g., systemic sclerosis and mixed connective tissue disease), hematological disorders (e.g., cryoglobulinemia), and endocrine disorders (e.g., hypothyroidism)^5^, which can cause more intense, frequent, and painful symptoms. Progressive RP may lead to severe functional disability, skin ulceration, and necrosis^6, 7^. Hence, it is essential to diagnose RP early to reduce the risk of functional impairment and improve the quality of life.

Primary or secondary RP diagnosis is mainly based on the patient’s subjective description of symptoms^8^. Comprehensive laboratory tests are ordered for further evaluation when the patient is suspected of having secondary RP. To assess vascular morphologic characteristics and evaluate the underlying disorders causing RP, diagnostic techniques such as nailfold capillaroscopy, digital photoplethysmography, or laser Doppler flowmetry can also be performed^9–12^. Previous studies have demonstrated the clinical significance of hand perfusion scintigraphy using radioisotopes for diagnosing RP, reporting sensitivity of up to 91.2% and specificity of 94%^13–17^. However, no studies have been conducted to evaluate the predictive value for therapeutic response using dynamic blood flow scintigraphy.

Therefore, the objective of this study was to investigate the clinical significance of quantitative parameters derived from ^99m^Tc-hydroxymethylene diphosphonate (HDP) dynamic blood flow scintigraphy to classify the disease severity and evaluate the predictive value of the therapeutic response in patients with RP.

## Materials and methods

### Patient selection

Our retrospective study was conducted between January 2014 and September 2020. It included 133 patients who reported RP symptoms and visited the rheumatology department. All patients received ^99m^Tc-HDP dynamic blood flow scintigraphy before starting treatment. The study population also met the following criteria: (1) patients had a history of cold-induced pallor of their hands followed by cyanosis, red flush, or painful condition; (2) patients who had not visited another hospital or had not been treated with these symptoms. Among them, we excluded those with RP in only feet and not hands (*n* = 16) and whose follow-up period was less than six months (*n* = 31). Six patients were excluded because of imaging loss. Ultimately, we enrolled 80 patients in this study.

RP was assessed through detailed history documentation, a physical examination, and serologic studies, including the erythrocyte sedimentation rate, anti-nuclear antibodies, anti-centromere antibodies, and anti-topoisomerase antibodies. Primary and secondary RP were diagnosed based on clinical and laboratory findings. The patients were divided into three groups according to the severity of RP symptoms. The mild group consisted of patients who had only hypersensitivity to cold exposure; the moderate group consisted of patients who showed color changes in their fingers upon cold exposure; and the severe group consisted of patients who experienced pain and/or abnormal sensations in their fingers, such as tingling or numbness.

All patients were instructed to take medication (e.g., calcium channel blocker and prostacyclin analogue) and/or undertake lifestyle modification, such as smoking cessation and/or avoidance of emotional and physical stress per their clinical presentation^18^. The patients were further categorized into two groups according to their therapeutic compliance and response i.e., good and poor. The patients with good compliance indicated those who adhered well to the treatment with lifestyle modification and/or regularly taking medication as prescribed. The poor compliance group consisted of patients who did not adhere to the treatment.

The corresponding physician evaluated the therapeutic response at least six months after the initial visit to our hospital based on clinical signs and/or symptoms during the follow-up period. The therapeutic response was defined as good when the patients and corresponding physician could observe that the frequency and intensity of RP symptoms were reduced. If the symptoms did not improve or if they worsened over time, it was considered a poor therapeutic response.

### Ethical statement

This study was conducted according to the guidelines of the Declaration of Helsinki and approved by the Institutional Review Board of Veterans Health Service Medical Center, Seoul, Republic of Korea (IRB No. 2022-12-024). The requirement for informed patient consent was waived by the ethics committee of Veterans Health Service Medical Center due to the retrospective nature of the study.

### Dynamic blood flow scintigraphy and image analysis

Although there have been various radiopharmaceuticals and protocols for Raynaud’s scintigraphy regarding the length of time allotted for immersion and recovery, the following method for one-hand chilling is widely used^13, 16^. ^99m^Tc-HDP dynamic blood flow scintigraphy was performed using a dual-head gamma camera with a high-resolution, low-energy parallel-hole collimator (E.CAM, v3.7, Siemens, Erlangen, Germany). At first, the patients were required to sit for at least 20 min in an examination room at 22 °C. Then, the patient’s hand that more predominantly manifested RP was selected for chilling. The selected hand was immersed in ice water (4 °C) for at least 2 min, followed by a recovery period of 15 min. During the recovery, the patients were instructed not to wipe or shake their hands. After hand chilling and recovery, the patients were asked to place both hands above the surface of the gamma camera detector with the fingers spread widely apart. Dynamic blood flow scintigraphy was acquired at a rate of 1 frame/3 s until 60 frames simultaneously with a bolus injection of 740 MBq of ^99m^Tc-HDP into the dorsal foot or lower leg vein.

Before measuring the quantitative parameters, two board-certified nuclear medicine physicians (J.Y. and M.C.) with more than 12 years of experience, who were strictly blind to the clinical information, interpreted the scintigraphy and reached a final consensus for each case. The results of their interpretation were recorded as positive or negative. The positive finding was defined as a definite discrepancy of radioactivity involving the chilled fingers and/or hand compared to the opposite fingers and/or hand. If no discrepancy was found, it was considered negative.

**Figure 1:**
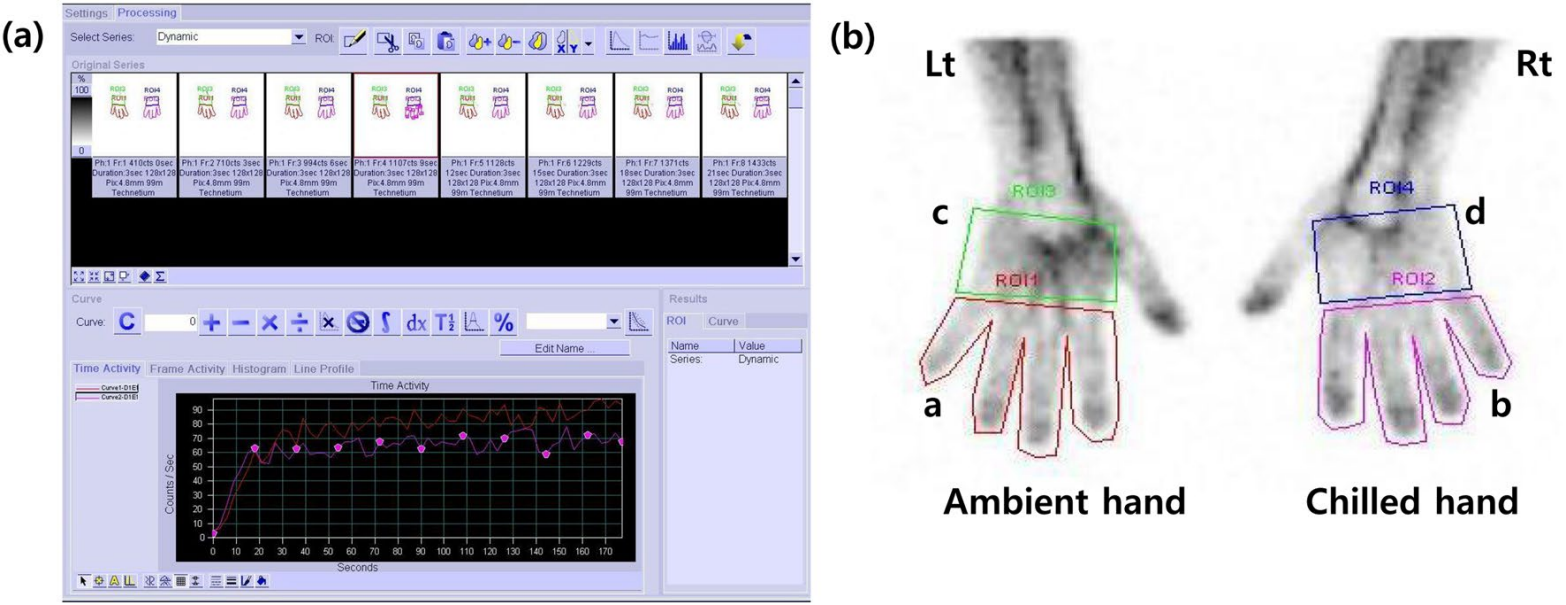
(**a**) ^99m^Tc-HDP blood flow scintigraphy in a patient with Raynaud’s phenomenon. (**b**) An example of regions of interest (ROI) for calculating chilled–ambient uptake ratios. Four ROIs were manually drawn around all fingers except the thumb (**a**,**b**) and palmar area (**c**,**d**) of both hands.

The regions of interest (ROIs) of all fingers (except the thumb) and palms of each hand on the summed image of the blood flow and static blood pool image were manually drawn by a physician (J.Y.) (Fig. 1). We obtained the radioactivity counts of ROIs on static blood pool images at 15 s and 30 s after radiopharmaceutical administration. The cumulative counts on the summed blood flow image for 180 s were also acquired using image analysis software (Syngo MI VA46B, Siemens, Erlangen, Germany). We respectively calculated three types of chilled–ambient ratios at each of the two times for further analysis using these counts within the ROIs as follows:

- Chilled–ambient finger ratio (CA_finger_): The CA_finger_ was calculated by dividing the counts of chilled fingers by the ambient fingers using the following formula: b/a
- Chilled hand–ambient hand ratio (CA_hand_): The CA_hand_ was measured by dividing the total counts of the chilled hand (fingers and palm) by those of the ambient hand at each time using the following formula: b + d/a + c
- Chilled finger–palm ratio (FPR) to ambient FPR ratio (CA_FPR_): The CA_FPR_ was also estimated using the following equation: b/d **/** a/c = bc/ad

The variables obtained at each time point were recorded as follows: CA_finger15s_, CA_finger30s_, CA_finger180s_, CA_hand15s_, CA_hand30s_, CA_hand180s_, CA_FPR15s_, CA_FPR30s_, CA_FPR180s_. The relationship of these quantitative variables with the clinical information, such as therapeutic response, was assessed to investigate the predictive significance of Raynaud’s scintigraphy.

### Statistical analysis

The continuous variables were recorded as the median with an interquartile range (IQR) or the mean ± standard deviation (SD) and a 95% confidence interval (CI) based on the given distribution. The Kolmogorov–Smirnov test was performed for normal distribution of all variables. The Mann–Whitney test was used to compare the quantitative parameters of scintigraphy between good and poor therapeutic responders when the continuous variables represented non-parametric results. If the variables indicated a normal distribution, an independent t-test was performed. Analysis of variance (ANOVA) was used to evaluate the differences between subgroups of RP severity and quantitative parameters. The inter-observer agreement of scintigraphy interpretation was measured using Fleiss’ kappa (κ). The κ-value ranges between − 1.0 and 1.0, with a value closer to 1.0 representing better reproducibility^19^.

To determine the optimal cutoff values of the quantitative scintigraphic parameters according to the therapeutic response, receiver operating characteristic (ROC) analysis was used. Next, we evaluated the predictive ability of the quantitative variables to identify the therapeutic response, including other clinical factors, using the chi-squared test and multivariate logistic regression analysis. The other clinical factors included the types of RP (primary vs. secondary), the intensity of RP, interpretation of scintigraphy, and therapeutic compliance. The statistical results were analyzed using MedCalc software package (Ver. 9.5, MedCalc Software, Mariakerke, Belgium), and the statistical significance was defined as a p-value less than 0.05.

## Results

### Patient characteristics

Table 1 shows the patient characteristics. Twenty-six patients (32.5%) were diagnosed with primary RP, and the remaining 54 (67.5%) were diagnosed with secondary RP based on clinical assessment using history documentation, physical examination, and laboratory findings. The primary RP group consisted of 20 male and six female patients. The secondary RP consisted of 18 male and 36 female patients. The patients with primary RP were younger (mean age ± SD; 37.9 ± 18.3 years) than those with secondary RP (mean age ± SD; 44.1 ± 16.1).

**Table 1:** Characteristics of patients.

	Primary RP (n = 26)	Secondary RP (n = 54)
Sex (male/female)	20/6	18/36
Age (years)		
Mean ± SD	37.9 ± 18.3	44.1 ± 16.1
Range	17.7–71.2	16.8–73.0
Follow-up period (months)		
Mean ± SD	36.7 ± 10.6	50.2 ± 17.0
Range	12.7–70.3	15.1–73.3
RP intensity		
Mild	6	6
Moderate	10	28
Severe	10	20
Therapeutic compliance		
Good	20	40
Poor	6	14
Therapeutic response		
Good	8	20
Poor	18	34
Scintigraphic finding		
Positive	22	38
Negative	4	16

*RP* Raynaud phenomenon.

According to the intensity of RP manifestations, 12 patients (15%) had mild RP, and 38 (47.5%) had moderate RP. The remaining 30 (37.5%) patients complained of severe symptoms on the date of their first visit to our hospital. Sixty patients (75%) had good therapeutic compliance, whereas 20 (25%) had poor compliance during the follow-up period (mean ± SD, 48.3 ± 18.0 months). Among them, 28 showed a good therapeutic response. Of the 52 patients with a poor therapeutic response, 40 complained of RP recurrence without considerable evidence of an improved condition. The remaining 12 reported having experienced more severe discomfort caused by RP.

### Results of ^99m^Tc-HDP dynamic blood flow scintigraphy

Sixty patients had positive scintigraphic findings, and 20 had negative findings. The corresponding κ-value was 0.9, being almost perfect agreement. Table 2 shows the results of the quantitative parameters from static scintigraphy at 15 s and 30 s and the cumulative counts of scintigraphy for 180 s concerning CA_finger_, CA_hand_, and CA_FPR_. Only CA_hand180s_ represented a normal distribution of continuous variables, which was the recorded mean ± SD. However, the remaining continuous variables were considered non-parametric tests, recorded median with IQR. Of all nine categories of continuous variables, CA_finger15s_, CA_finger30s_, CA_finer180s_, CA_hand15s_, CA_hand30s_, CA_hand180s_, CA_FPR30s_, and CA_FPR180s_ had significant correlation with a therapeutic response (all p < 0.05), as those parameters decreased in the patient group with a poor therapeutic response. In the patient groups of the two categories divided into primary and secondary RP, there were statistical differences in only those with early static blood counts, such as CA_finger15s_ (p = 0.016) and CA_hand15s_ (p = 0.025). The ANOVA indicated that the intensity of RP was statistically dependent on all of the quantitative parameters (all p < 0.05). The patients with more severe RP symptoms had significantly lower values than those with less severe RP symptoms (Table 3).

**Table 2:** Correlation between quantitative parameters from scintigraphy and clinical features.

	Therapeutic response		P value	Types of RP		p-value
	good	Poor		Primary	secondary	
CA_finger15s_	1.40, 0.44–4.13	0.28, 0.10–0.73	0.008*	0.63, 0.26–3.02	0.14, 0.07–0.79	0.016*
CA_finger30s_	1.65, 0.70–4.50	0.50, 0.30–0.92	0.001*	0.71, 0.38–1.73	0.57, 0.30–1.06	0.444
CA_finger180s_	1.12, 0.62–2.56	0.53, 0.41–0.81	0.004*	0.70, 0.51–1.49	0.56, 0.43–0.86	0.279
CA_hand15s_	0.79, 0.45–2.27	0.31, 0.21–0.58	0.005*	0.58, 0.34–1.61	0.25, 0.14–0.58	0.025*
CA_hand30s_	0.91, 0.48–1.62	0.53, 0.42–0.64	0.018*	0.64, 0.48–1.21	0.50, 0.45–0.68	0.194
CA_hand180s_	0.98 ± 0.46	0.66 ± 0.26	0.031*	0.83 ± 0.40	0.65 ± 0.27	0.109
CA_FPR15s_	1.28, 0.94–3.67	0.87, 0.34–1.67	0.061	1.26, 0.61–3.55	0.66, 0.25–1.32	0.051
CA_FPR30s_	1.74, 1.12–4.68	0.98, 0.51–1.64	0.004*	1.37, 0.81–2.24	1.08, 0.55–1..25	0.231
CA_FPR180s_	1.58, 0.97–2.21	0.98, 0.66–1.27	0.002*	1.27, 0.81–1.89	0.97, 0.70–1.17	0.292

*CA*_*finger*_ chilled fingers to ambient fingers ratio, *CA*_*hand*_ chilled hand to ambient hand ratio, *CA*_*FPR*_ chilled finger-to-palm ratio (FPR) to ambient FPR ratio, *RP* Raynaud phenomenon.

*p < 0.05.

**Table 3:** Correlation between quantitative parameters from scintigraphy and intensity of RP.

	RP intensity			p-value
	Mild (n = 12)	Moderate (n = 38)	Severe (n = 30)	
CA_finger15s_	2.11, 0.99–3.00	0.93, 0.67–3.86	0.12, 0.08–0.36	< 0.001*
CA_finger30s_	1.65, 1.14–6.20	0.81, 0.57–1.78	0.34, 0.21–0.54	< 0.001*
CA_finger180s_	1.37, 1.08–2.67	0.81, 0.63–1.55	0.44, 0.30–0.53	< 0.001*
CA_hand15s_	1.54, 0.77–2.47	0.58, 0.30–1.61	0.24, 0.19–0.46	0.002*
CA_hand30s_	1.27, 0.91–1.78	0.64, 0.55–1.21	0.44, 0.40–0.50	< 0.001*
CA_hand180s_	1.22 ± 0.48	0.84 ± 0.31	0.51 ± 0.11	< 0.001*
CA_FPR15s_	1.47, 1.26–3.21	1.64, 0.90–3.67	0.34, 0.21–0.79	< 0.001*
CA_FPR30s_	2.38, 1.08–4.68	1.34, 1.12–2.47	0.55, 0.78–1.12	0.002*
CA_FPR180s_	1.75, 0.97–2.21	1.27, 0.97–1.80	0.71, 0.48–0.89	0.001*

*CA*_*finger*_ chilled fingers to ambient fingers ratio, *CA*_*hand*_ chilled hand to ambient hand ratio, *CA*_*FPR*_ chilled finger-to-palm ratio (FPR) to ambient FPR ratio, *RP* Raynaud phenomenon.

*p < 0.05.

The reverse scintigraphic finding, defined as an increased blood flow of the chilled hand rather than the ambient side, was observed in 18 patients. In the reverse scintigraphic findings, 12 patients had a good therapeutic response. Meanwhile, 46 of the remaining 62 patients with non-reverse scintigraphic findings had a poor therapeutic response (p = 0.053).

**Figure 2:**
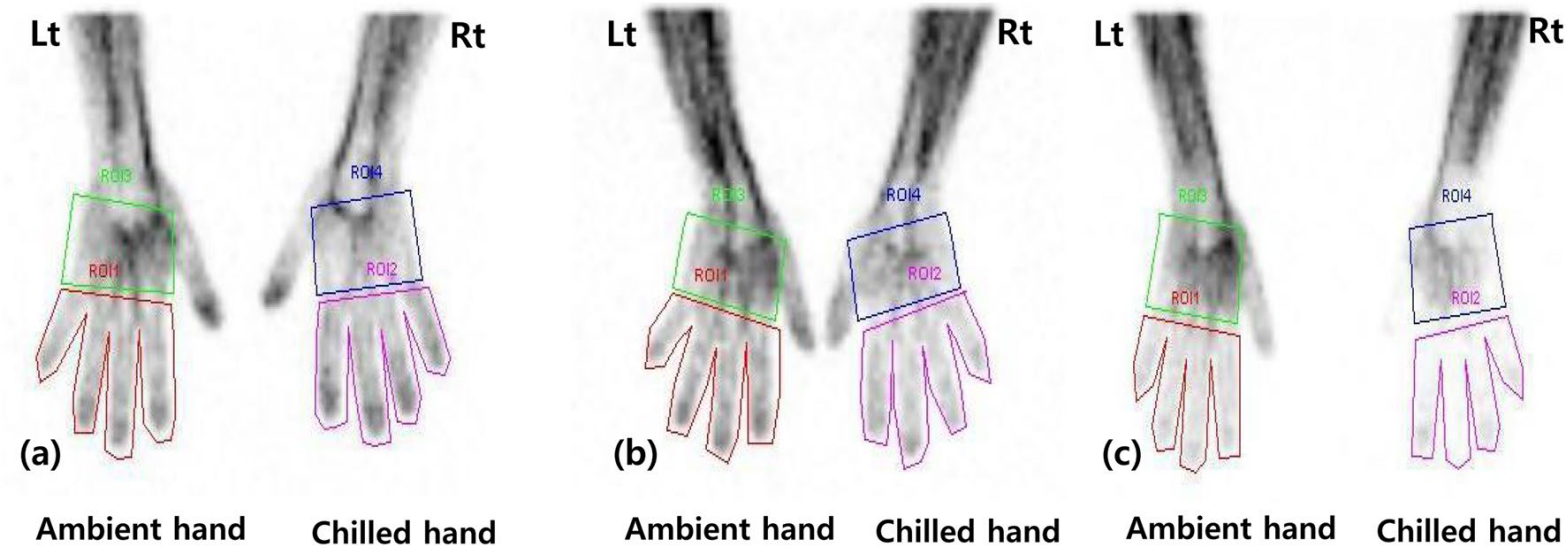
Representative dynamic blood flow scintigraphy images with different therapeutic responses. (**a**) Scintigraphy of 38-year-old woman with mild RP in both hands (dominant in right hand). This image showed no significantly reduced radioactivity uptake in the chilled hand. The chilled–ambient finger ratio at 30 s (CA_finger30s_) was 1.16. She had a good therapeutic response in the follow-up period. Her symptoms improved significantly after one year of treatment. (**b**) Scintigraphy of 45-year-old woman with moderate RP only in the right hand. After cold stimulation, the radioactivity uptake of the right fingers was observed to be somewhat reduced, and CA_finger30s_ was 0.89. She appropriately underwent lifestyle modification and medication during follow-up, but the therapeutic response was poor. (**c**) Scintigraphy of 42-year-old woman with moderate RP in both hands (dominant in right hand). The right fingers had decreased blood pool activity compared with the opposite fingers of the hand not undergoing cold stimulation. The CA_finger30s_ was 0.74. She also had a poor therapeutic response in the follow-up period.

ROC analyses suggested the optimal cutoff values of each quantitative parameter derived from the chilled–ambient ratios for predicting the therapeutic response. The results of univariate analysis using the chi-squared test are listed in Table 4. Significant differences are shown between good and poor therapeutic responses in terms of RP intensity (p = 0.003) and quantitative parameters (all p < 0.05, except only CA_FPR15s_). There were no significant differences between those in patient groups according to therapeutic compliance (p = 0.126), etiology of RP (p = 0.972), and the interpreted results of scintigraphy (p = 0.126). After multivariate analysis, only CA_finger30s_ (p = 0.002) showed independent predictability of therapeutic response in patients with RP (Table 4). Representative cases with different therapeutic responses are depicted in Fig. 2.

**Table 4:** Univariate and multivariate analyses for predicting therapeutic response.

Univariate	Cutoff value	Therapeutic response		p-value
		Good (n = 28)	Poor (n = 52)	
CA_finger15s_	≤ 0.73	8	40	0.008*
CA_finger30s_	≤ 1.10	10	46	0.002*
CA_finger180s_	≤ 0.54	2	28	0.011*
CA_hand15s_	≤ 0.58	8	40	0.008*
CA_hand30s_	≤ 0.84	12	46	0.007*
CA_hand180s_	≤ 0.79	12	44	0.017*
CA_FPR15s_	≤ 0.91	6	30	0.062
CA_FPR30s_	≤ 0.90	2	26	0.004*
CA_FPR180s_	≤ 0.89	2	28	0.002*
RP intensity	Mild	10	2	0.003*
	Moderate	14	24	
	Severe	4	26	
Therapeutic compliance	Good	26	34	0.126
	Poor	2	18	
Types of RP	Primary	8	18	0.972
	Secondary	20	34	
Scintigraphic finding	Positive	16	44	0.126
	Negative	12	8	

Multivariate	Cutoff value	Therapeutic response		p-value
		Odds ratio	95% CI	
CA_finger30s_	≤ 1.10	13.80	2.716–70.127	0.002

*CA*_*finger*_ chilled fingers to ambient fingers ratio, *CA*_*hand*_ chilled hand to ambient hand ratio, *CA*_*FPR*_ chilled finger-to-palm ratio (FPR) to ambient FPR ratio, *RP* Raynaud phenomenon, *CI* confidence interval.

*p < 0.05.

## Discussion

For patients with either primary or secondary RP, the treatment is focused on the underlying pathophysiologic disorders and the severity of the clinical manifestations^18, 20^. Most patients with RP are typically treated with conservative management, including avoidance of exacerbating factors such as cold or stress exposure, smoking cessation, and maintaining warmth of the body parts showing RP. However, these lifestyle modifications are only temporarily effective; they often only result in partial resolution of RP. In numerous medical institutions, pharmacologic treatment (e.g., calcium channel blockers, alpha one receptor antagonists, renin-angiotensin system mediators, phosphodiesterase inhibitors, prostacyclin analogues, endothelin inhibitors) has been applied to alleviate the frequency and intensity of symptoms rather than complete treatment of the underlying disorder. Among them, the most commonly prescribed medications are calcium channel blockers (e.g., Nifedipine) and prostacyclin analogues (e.g., Iloprost). Although previous studies suggested that pharmacologic approaches could be efficacious for treatment, some patients showed only minimal benefit or experienced adverse side effects, often requiring medication discontinuation^21, 22^. Based on these factors, we assumed that evaluating the objective predictive methods for therapeutic response at the initiation of treatment is clinically important.

In the present study, we demonstrated the predictive significance of ^99m^Tc-HDP early dynamic blood flow scintigraphy using the one-hand chilling method for therapeutic response during the follow-up period. In cases with lower values of chilled–ambient ratios, we found that the effect of therapeutic responses decreased more notably than those in the other groups. In particular, for those with a value lower than 1.10 of CA_finger30s_, it was an independent predictor factor for patients having a poor therapeutic response. Additionally, we found that some patients with RP showed higher blood flow in the chilled hand compared to the ambient side, demonstrating a reverse effect after cold exposure. These findings were also noted in a previous study^14^. Patients with reversely increased blood flow after chilling tended to have a better therapeutic response than patients with decreased blood flow. However, the difference was not statistically significant. We assumed this result could have occurred because erythema is known as the reperfusion phase of RP.

We also identified the efficacy of dynamic blood flow scintigraphy in differentiating between primary and secondary RP using the chilled–ambient ratios obtained in 15 s, but not 30 s or 180 s, which means that there were only differences of blood flow in very early time point between primary and secondary RP. This result was considered a feature of the internal distribution of ^99m^Tc-HDP, which enters the vascular system immediately after bolus injection and can indicate hemodynamic changes in the microcirculation of peripheral body parts. Based on this theory, we assumed that functional changes of the vascular system in primary RP might not appear in the very early time after cold exposure. However, it is considered difficult to meaningfully distinguish primary and secondary RP with chilled–ambient ratios, except during very early periods. A previous study^23^ showed similar results, with no significant difference in the radioactivity count of finger–palm ratios between primary and secondary RP. However, there were some differences in materials and methods used compared to our study’s.

The present study had several limitations. First, a small number of patients were enrolled. Furthermore, we had to consider a selection bias because of the clinical characteristics of RP. Many patients with RP do not visit clinical institutes because their symptoms may not affect their quality of life and can improve with time. The symptoms may be alleviated by lifestyle modification^24–26^. Moreover, the exclusion criteria comprised a relatively large number of patients with a follow-up period of less than six months. Many patients did not seek subsequent physician care because their episodes were not severe and might not have occurred only in cold weather. Second, this retrospective design was partly subjective concerning reviewing the clinical information on therapeutic outcomes and compliance. However, the clinical chart review was performed blindly for the scintigraphic findings. Third, we did not consider the onset age and duration of RP. A previous report suggested that the age of the patient at the onset of RP and the duration were correlated with the radioactivity counts of the patient’s fingers and palm^27^. A critical limiting factor is the lack of validated objective methods for assessing the semiquantitative variables. Therefore, further study is needed to suggest standard variables for imaging analysis with many subjects.

Despite these limitations, this study is clinically essential for investigating the predictive value of therapeutic response using ^99m^Tc-HDP dynamic blood flow scintigraphy in patients with RP. Using the objective predictive factors of blood flow scintigraphy, a clinician can strongly encourage patient therapeutic compliance when a good therapeutic response is expected before treatment. This involves explicitly strict adherence to treatment, including lifestyle modification and consistent medication use as directed. If a poor therapeutic response is predicted, the clinician can consider increasing the medication dosage or adding other medications while conducting close clinical follow-up.

## Conclusion

Raynaud’s scintigraphy with one-hand chilling method can be used to predict whether RP patients will have good or poor response to treatment during the follow-up period. However, as mentioned above, there has been a considerable variation in the protocols performed in Raynaud’s scintigraphy according to radiopharmaceuticals, cold stimulation, and ROI methods. In the future, standard protocols for Raynaud’s scintigraphy should be established for patients with RP.

## Data Availability

All the data relevant to this study are included in the manuscript, tables and figures. Additional data will be provided upon request.
